# Frequency of *Trichomonas vaginalis, Candida sp and Gardnerella vaginalis* in cervical-vaginal smears in four different decades

**DOI:** 10.1590/S1516-31802001000600004

**Published:** 2001-11-01

**Authors:** Sheila Jorge Adad, Rodrigo Vaz de Lima, Zahir Tannous Elias Sawan, Maria Letícia Gobo Silva, Maria Azniv Hazarabedian de Souza, João Carlos Saldanha, Vera Alice Aguiar Falco, Afife Hallal da Cunha, Eddie Fernando Cândido Murta

**Keywords:** Vaginitis, Cervical-vaginal cytopathology, Trichomonas vaginalis, Candida sp, Gardnerella vaginalis, Vaginite, Citopatologia cérvico-vaginal, Trichomonas vaginalis, Candida sp, Gardnerella vaginalis

## Abstract

**CONTEXT:**

Vaginitis is one of theprincipal motives that lead women to seek out an obstetrician or gynecologist. Bacterial vaginosis, candidiasis and trichomoniasis are responsible for 90% of the cases of infectious vaginitis.

**OBJECTIVE:**

To verify the frequency of the three main causative agents of vaginitis, Trichomonas vaginalis, Candida sp and Gardnerella vaginalis, in four different decades (1960's, 1970's, 1980's and 1990's).

**DESIGN:**

Retrospective.

**PLACE:**

A tertiary referral center.

**PARTICIPANTS:**

Patients attended to as gynecology and obstetrics out patients at the Faculdade de Medicina do Triângulo Mineiro during the years 1968, 1978, 1988, 1998, taken as samples of each decade.

**MAIN MEASUREMENTS:**

Diagnoses of infection by Trichomonas vaginalis, Candida sp and Gardnerella vaginalis were gathered from 20,356 cervical-vaginal cytology tests on patients attended to as gynecology outpatients at Faculdade de Medicina do Triângulo Mineiro during the years 1968, 1978, 1988, 1998, representing the four decades. The results were grouped according to the age group of the patients: under 20, between 20 and 29, between 30 and 39, between 40 and 49, and 50 or over. Statistical analysis was done via the chi-squared(Mantel-Haentzel)test with a significance level of 5%.

**RESULTS:**

In 1968 infections by Trichomonas vaginalis and Candida sp were diagnosed in 10% and 0.5% of the cytology tests and in 1978, 5.1%and 17.3%, respectively(P < 0.0001). Infection by Gardnerella vaginalis could only be evaluated in the latter two decades. In 1988, 19.8% of the women had positive tests for Gardnerella vaginalis, which was the most frequent agent in that year, diminishing in the subsequent decade to 15.9% (P < 0.0001). Candidiasis was the most frequent infection in 1998, detected in 22.5%of the tests (P < 0.0001). In a general manner, all the infections were most frequent among younger patients, especially those aged under 20, in all decades, whereas infections were least frequent among patients aged 50 or over(P< 0.05).

**CONCLUSION:**

There was a reduction in the frequency of cervical-vaginal infection byTrichomonas vaginalis and an increase in the frequency of Candida sp over the four decades studied. All the infections were most frequent in patients aged under 20 years.

## RESUMO

**CONTEXTO:**

A vaginite é uma das principais causas que leva a mulher procurar um ginecologista ou obstetra. A vaginose bacteriana, a candidíase e a tricomoníase são responsáveis por 90% dos casos de vaginite infecciosa.

**OBJETIVO:**

Verificar a freqüência dos três principais agentes causadores de vaginite, *Trichomonas vaginalis, Candida sp* e *Gardnerella vaginalis* em quatro diferentes décadas (60, 70, 80 e 90).

**TIPO DE ESTUDO:**

Estudo retrospectivo. **LOCAL:** Centro Terciário de Referência.

**PARTICIPANTES:**

Pacientes atendidas no ambulatório de Ginecologia e Obstetrícia da Faculdade de Medicina do Triângulo Mineiro, durante os anos de 1968, 1978, 1988, 1998, como amostras de cada década.

**VARIÁVEIS ESTUDADAS:**

Foram levantados os diagnósticos de infecção por *Trichomonas vaginalis, Candida sp* e *Gardnerella vaginalis* em 20.356 citologias cérvico-vaginais de pacientes atendidas no ambulatório de Ginecologia, da mesma Instituição, durante os anos de 1968, 1978, 1988, 1998, os quais representariam as quatro diferentes décadas. Os resultados foram agrupados de acordo com a faixa etária da paciente: menos de 20 anos, entre 20 e 29 anos, entre 30 e 39 anos, entre 40 e 49 anos e 50 anos ou mais. A análise estatística foi feita através do teste do qui-quadrado (Mantel-Haentzel) com nível de significância de 5%.

**RESULTADOS:**

Em 1968, a infecção por *Trichomonas vaginalis* e *Candida sp* foram diagnosticadas em 10% e 0,5% das citologias e em 1978, 5,1% e 17,3%, respectivamente (P < 0,0001). A infecção por *Gardnerella vaginalis* só pôde ser avaliada nas duas últimas décadas. Em 1988, 19,8% das mulheres possuíam exames positivos para esse agente que foi o mais freqüente nesse ano; na década seguinte a freqüência de Gardnerella diminuiu para 15,9% (P < 0,0001). No ano de 1998, a candidíase foi a infecção mais freqüente, detectada em 22,5% dos exames (P < 0,0001). De modo geral, todas as infecções foram mais freqüentes nas pacientes mais jovens, em especial abaixo de 20 anos, em todas as décadas e, ao contrário, menos freqüentes em pacientes com 50 anos ou mais (P < 0,05).

**CONCLUSÃO:**

Houve uma diminuição na freqüência de infecção cérvico-vaginal por *Trichomonas vaginalis* e um aumento na freqüência de *Candida sp* ao longo das quatro décadas estudadas. Todas as infecções foram mais freqüentes nas pacientes abaixo de 20 anos.

## INTRODUCTION

Vaginitis, whether infectious or not, constitutes one of the most common problems in clinical medicine, and it is one of the main motives that lead women to seek out an obstetrician or gynecologist.^1^ Bacterial vaginosis, candidiasis and trichomoniasis are responsible for 90% of the cases of infectious origin.^2^

Bacterial vaginosis is characterized by the substitution of the vaginal flora, normally dominated by lactobacilli, by a complex and abundant flora of strictly or optionally anaerobic bacteria that are normally found in the vagina (*Gardnerella vaginalis, Bacteroides sp, Peptostreptococcus, Mobiluncus sp)*.^3^ Abundant foul-smelling vaginal secretions are the typical symptom of infection by *Gardnerella vaginalis*.^4^ The symptomatic infection by *Candida sp* arises when there is an excessive proliferation of this microorganism in the vaginal flora, ceasing its colonization and starting to achieve outright adherence to the vaginal cells, consequently causing infection.^5^ The patient presents thick, fetid vaginal secretions with a granular appearance and an itchy vulva. The vagina becomes hyperemic and the vulva erythematous, and there may be excoriation and dyspareunia.^6^ *Trichomonas vaginalis* is a flagellate protozoan considered to be sexually transmittable and related to low socioeconomic levels.^7^ Typically, a patient with trichomoniasis presents intense frothy yellow-greenish vaginal discharges, irritation and pain in the vulva, perineum and thighs, and dyspareunia and dysuria.^5^

Diverse studies performed with the objective of establishing the frequency of the most common infectious agents for vaginitis have shown widely varying results. The indices found for *Gardnerella vaginalis* have varied between 8% and 75%, *Candida albicans* has presented rates between 2.2% and 30%, and *Trichomonas vaginalis* between zero and 34%.^8-17^

Analysis of the frequency of the causative agents for infectious vaginitis over different decades may reflect the modifications in habits and living conditions experienced by a given population, as well as the introduction of new technologies and the frequent use of pharmacological agents.

This study was made with the objective of comparing the frequency of the main causative agents of vaginitis, *Trichomonas vaginalis, Candida sp* and *Gardnerella vaginalis* over the last four decades.

## METHODS

The study was performed in the Cytopathology Service of the Faculdade de Medicina do Triângulo Mineiro. Diagnoses of infection by *Trichomonas vaginalis, Candida sp* and *Gardnerella vaginalis* were gathered from 20,356 cervical-vaginal cytology tests on patients attended to as gynecology outpatients at Faculdade de Medicina do Triângulo Mineiro during the years 1968, 1978, 1988 and 1998, representing the four different decades.

The smears from these reports were fixed in ordinary ethyl alcohol and stained using Papanicolaou's method.

**Figure 1:**
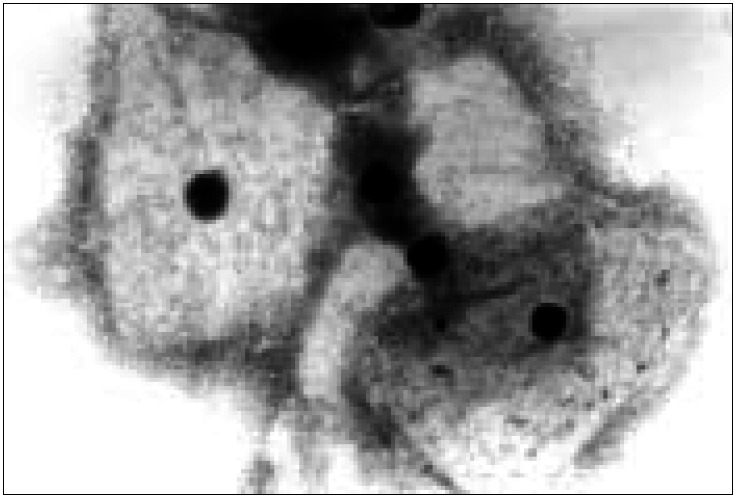
Smear presenting "clue cells". Note the bacteria not only covering the surface of the squamous cells but also smudging the cytoplasm limit (Papanicolaou staining; 1000x).

The readings were done by doctors specializing in cytopathology, trained by the cytopathologist who initiated the use of the Papanicolaou test in our Institution, utilizing the same diagnostic criteria in relation to *Candida* and *Trichomonas*, over the four decades. With regard to *Gardnerella vaginalis*, in the first two decades it was not separately diagnosed and it was included in the group of mixed bacterial flora. In the latter two decades, the diagnosis of *Gardnerella vaginalis* was made on the basis of a finding of "clue-cells" (Figure 1). For this reason, the frequency of *Gardnerella* was only evaluated for the latter two decades. "Clue cells" are squamous cells covered with coccobacilli whose cytoplasmic borders are presented as smudged (Figure 1).

**Figure 2:**
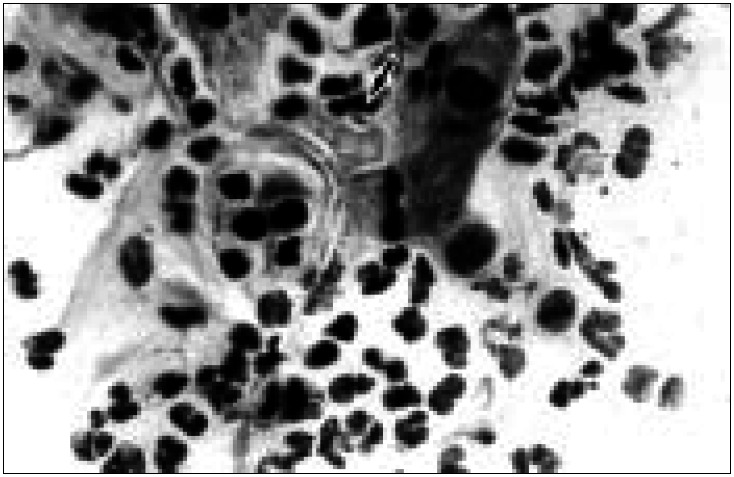
Pseudo-hyphae and spores of Candida sp*; observe the clear halo around the spore (arrow) and the filamentous structures (Papanicolaou staining; 1000x).*

*Candida sp* was diagnosed when pseudo-hyphae were seen, weakly stained with eosin or sometimes with hematoxylin, and/or small spores (diameters of 2-4 mm), stained pale pink (Figure 2).

**Figure 3:**
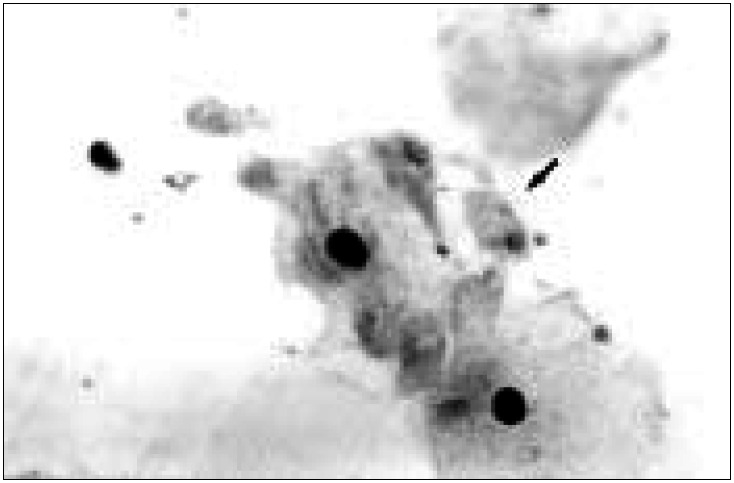
Trichomonas vaginalis *is an ovoid or triangular organism (arrow), generally measuring between 8 and 20 mm, with a vesicular nucleus, lightly stained by hematoxylin (Papanicolaou staining; 1000x).*

*Trichomonas vaginalis* was diagnosed when a unicellular organism of ovoid or rounded shape was viewed (diameter of 8-20 mm), with pallid or grayish cytoplasm. It could have eosinophilic granules at its center and a vesicular or crescent-shaped nucleus, lightly stained by hematoxylin (Figure 3).

The results were grouped according to the age groups of the patients: under 20, between 20 and 29, between 30 and 39, between 40 and 49, and 50 or over.

Statistical analysis was done via the chi-squared (Mantel-Haentzel) test. The significance level considered for all the tests was 5% (P < 0.05).

## RESULTS

A total of 20,356 reports were analyzed, of which 880 (4.3%) were from the year 1968, 3026 (14.9%) from 1978, 6825 (33.5%) from 1988, and 9625 (47.3%) from 1998.

In all these decades, the age group that most frequently underwent the Papanicolaou test at Faculdade de Medicina do Triângulo Mineiro was that of women between 20 and 29 years, representing 31.7% of the total. However, while in 1968 41.1% of the tests were on patients in this age group, in 1998 only 26.3% were in this age group.

**Figure 4:**
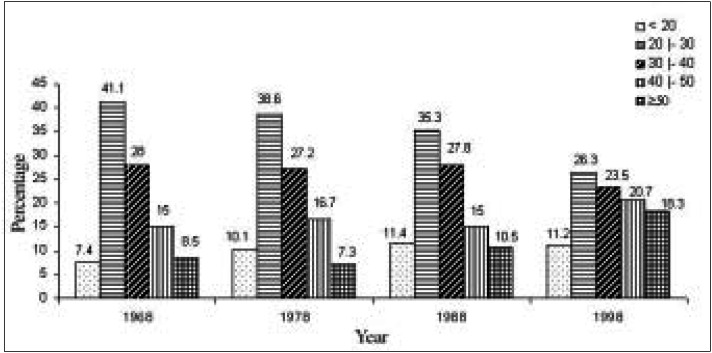
Distribution of patients in different decades, according to age group.

The inverse was observed (Figure 4) in the group of patients aged between 40 and 49 years (15% in 1968, rising to 20.7% in 1998), the group aged 50 years and over (from 8.5% in 1968 to 18.3% in 1998), and the group aged under 20 years (from 7.4% in 1968 to 11.2% in 1998), with these differences being statistically significant.

**Figure 5:**
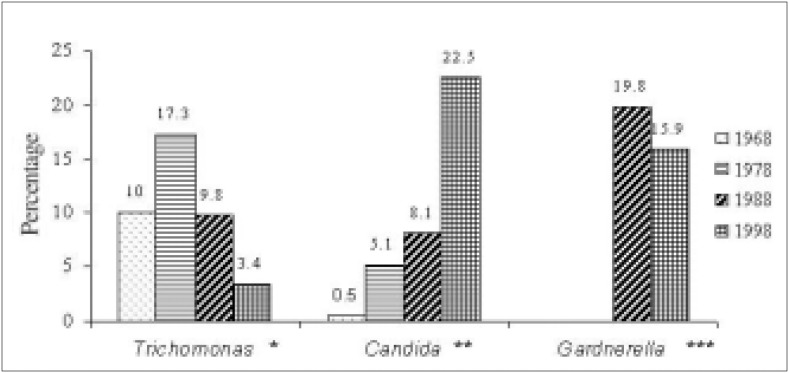
Frequency of Trichomonas, Candida *and* Gardnerella *in cervical vaginal cytologies from four different decades.*

Figure 5 shows the frequency of *Trichomonas vaginalis, Candida sp* and *Gardnerella vaginalis* in the 4 different years. It can be noted that in 1968 trichomoniasis was the most frequent infection, diagnosed in 10% of the examinations, while candidiasis was detected in only 0.5%. In 1978, candidiasis was diagnosed in 5.1% of the tests, although trichomoniasis was still the most frequent infection, detected in 17.3%. In 1988, 19.8% of the women had positive tests for *Gardnerella vaginalis*, which was the most frequently diagnosed agent in that year. In 1998, candidiasis was the most frequent infection, diagnosed in 22.5% of the tests.

*, **, *** P< 0.0001

Comparing now the frequency of each agent over the decades, it can be seen that there was between 1969 and 1978 an increase in the frequency of infection by *Trichomonas vaginalis*, although in the subsequent decades the inverse occurred, and these differences were statistically significant. In relation to the frequency of *Candida sp*, there was a significant increase from 1968 to 1998. Regarding *Gardnerella vaginalis*, a significant reduction was observed between the two decades that could be evaluated (1988 and 1998).

**Figure 6:**
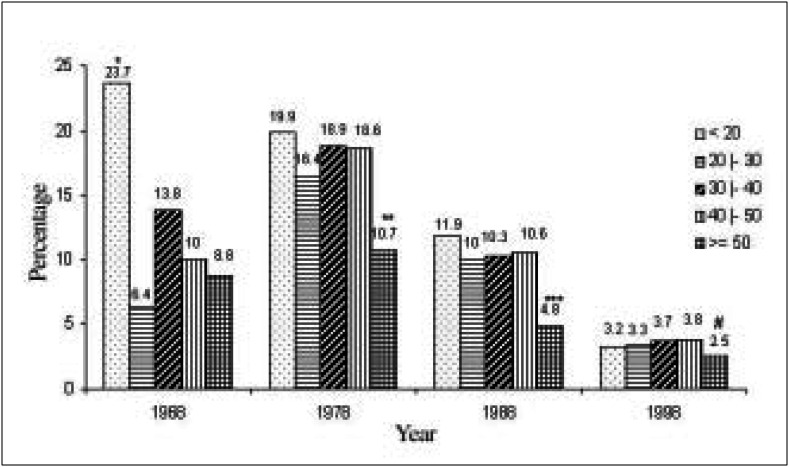
Distribution of patients with Trichomonas vaginalis *in cervical-vaginal cytologies from four different decades, according to age group*

**Figure 7:**
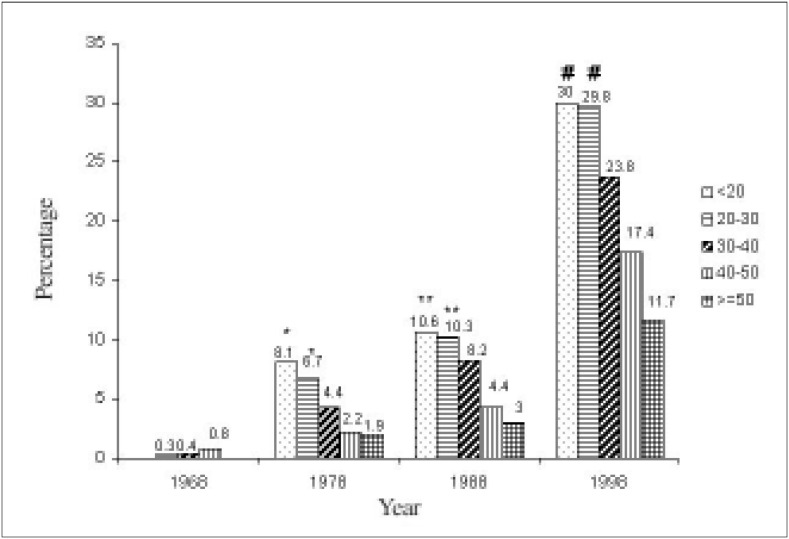
Distribution of patients with Candida sp in cervical-vaginal cytologies in four different decades, according to age group.

In Figure 6, it can be seen that in the year 1968, *Trichomonas vaginalis* was most frequent in the group aged under 20. In the years 1978 and 1988, this agent was statistically more frequent in the 30 to 39 and 40 to 49 age groups, in relation to the over-50 age group. In Figure 7, it can be observed that in the year 1968, there was no statistical difference in the frequency of *Candida sp* in the different age groups. From 1978 onwards, there was a statistically significant difference between the extremes of the age groups, with greatest frequency of *Candida sp* in women aged less than 30 years.

*P<0.05 compared with the other age groups in 1968.

**P<0.05 compared with the other age groups in 1978.

***P< 0.05 compared with the other age groups in 1988.

***#P< 0.05 compared with the 30 to 39 and 40 to 49 age groups.***

*P< 0.05 in relation to the 30 to 39, 40 to 49 and over 50 age groups.

**P< 0.05 in relation to the 30 to 39, 40 to 49 and over 50 age groups.

***#P<0.05 in relation to the 30 to 39, 40 to 49 and over 50 age groups.***

**Figure 8:**
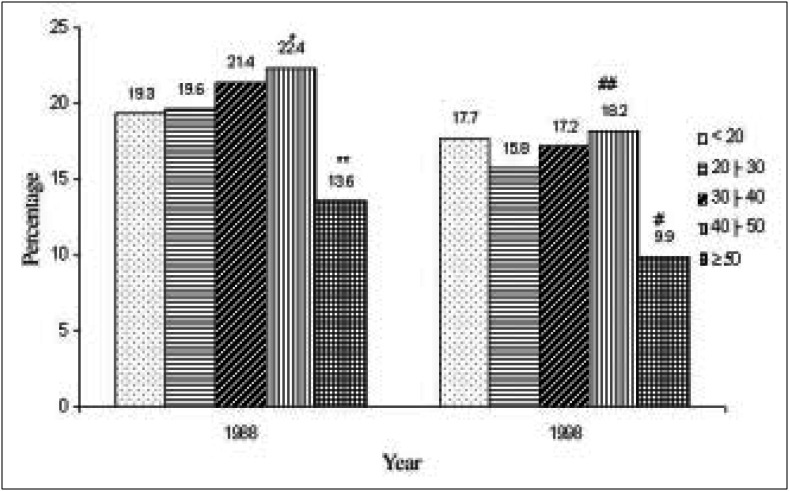
Distribution of patients with Gardnerella vaginalis *in cervical-vaginal cytologies in two different decades, according to age group.*

Regarding the frequency of *Gardnerella vaginalis* in the different age groups, it can be seen in Figure 8 that this agent was most frequent in women aged between 40 and 49 years. This was statistically significant in relation to women aged 50 years of over, and in relation to the 20 to 29 age group. Women aged 50 or over had the lowest frequency of *Gardnerella vaginalis* in relation to the other age groups.

*P< 0.05 compared with the 20 to 29 age group.

**P<0.05 compared with the other age groups.

***#P<0.05 compared with the other age groups.***

***##P<0.05 compared with the 20 to 29 age group.***

Detailed analysis was only done in relation to these three infections, as these are responsible for more than 90% of vaginal infection cases. Among the 20,356 examinations there were only 21 cases (0.01%) of Herpes virus and 16 (0.008%) of Actinomyces. In relation to the cytological diagnosis of infection by human papillomavirus (HPV), indicated after 1978, we found a frequency of 5.4% in 1988 and 2.8% in 1998.

With regard to the Papanicolaou classes, a classification we used before 1988, we found 2.4% with class III (mild, moderate or severe dysplasias) or class IV (carcinoma *in situ*) in 1968 and 2.9% in 1978. Converting the diagnoses of cervical intraepithelial neoplasia (CIN) grades I, II and III into the old Papanicolaou classes, we have 2.2% with classes III or IV in 1988 and 3.6% in 1998, providing evidence of an increase in the frequency of CIN in the past decade.

## DISCUSSION

In relation to the frequencies of the different vaginal pathogens, high indices of infection by *Candida sp* were found in the past decade (22.5% in 1998) and low indices of trichomoniasis (3.4% in the same period). These data are compatible with those in other publications. Hart,^11^ studying 5,365 women independent of their clinical condition between 1988 and 1991, found trichomoniasis in 1.8%, bacterial vaginosis in 13.7% and candidiasis as the main agent in 17.6% of the patients. Ray et al.,^10^ studying 100 women with vaginitis and 50 asymptomatic women, found *Trichomonas vaginalis* in 11.1%, *Candida albicans* in 30% and *Gardnerella vaginalis* in 31% of the first group, while in the second group these values were 0%, 14% and 22%, respectively. Toloi et al.^15^ analyzed 133 patients and found *Candida* in 26%, *Gardnerella* in 8% and 0% for *Trichomonas.*

Kent,^1^ studying the epidemiology of the three main causes of vaginitis in the USA and Scandinavia, observed that the frequency of *Trichomonas vaginalis* has diminished markedly in both regions over recent years. This was also observed in the present study (17.3% in 1978 down to 3.4% in 1998), raising the hypothesis that this is a possible consequence of the introduction of metronidazole in medical therapeutics and the improvement in conditions of hygiene. However, in other countries like Venezuela, the frequency of infection by *Trichomonas* still remains very high, detected in 24.6% of the 630 examinations made in a university hospital.^14^

Kent^1^ reported a fall in the frequency of *Candida albicans* in the USA and a stabilization of infection by this agent in Scandinavia, while in our data, infection by *Candida* has presented a large increase over the last decade (8.1% in 1988 to 22.5% in 1998). It is possible that the increase is a result of the use of oral contraceptives, and also the use of hormone replacement therapy, an increase in the number of immunologically compromised patients (HIV, cortical therapy), changes in sexual habits (correlation with clinical sexually transmittable diseases), and from clothing and the abusive use of antibiotics.

Regarding *Gardnerella*, several works have identified it as the leading vaginal infection in the countries researched. In a cytology clinic in Ibadan, Nigeria, *Gardnerella vaginalis* was found in 9.8% of the examinations,

*Trichomonas vaginalis* in 2.5% and *Candida albicans* in 2.2%,^8^ while in a clinic for sexually transmittable diseases in Nairobi, Kenya, the frequency of these pathogens was 75%, 34% and 24%, respectively.^9^

In a laboratory in Belo Horizonte, Brazil, in which tests on private patients predominated, the frequency of infections was much lower: *Gardnerella* 14.1%, *Candida* 6.9% and *Trichomonas* 1.1%.^17^ It is probable that these lower indices are related to the socioeconomic power of the patients.^17^ Oyarzún et al.,^12^ in Chile, have detected *Candida* in 16.8%, *Gardnerella* in 11.1% and *Trichomonas* in 1.6%. These authors made a diagnosis of bacterial vaginosis when they encountered at least three of the following four criteria: homogenous vaginal discharge, vaginal pH > 4.5, an odor of fish when the vaginal secretion was alkalinized, and the presence of "clue cells".^12^

It can be seen in the present work that *Gardnerella* has frequently been diagnosed over recent decades, although it was supplanted in frequency by *Candida* in the year 1998. The absence of diagnosed cases of *Gardnerella vaginalis* until 1978 is due to the fact that until then this diagnosis was not routinely made via cytological examination, with this agent being grouped within the mixed bacterial flora. Successive works in the 1980's proved that the finding of "clue-cells" in the cytological examination presented a high correlation with a positive culture for *Gardnerella vaginalis*.^18,19^ From this time onwards, cytopathology services have generally considered a diagnosis of *Gardnerella* when "clue-cells" are encountered. The study made by Oyarzún et al^12^ corroborates this practice, as they observed that the finding of "clue cells" had a 100% sensitivity and a 96% specificity for the diagnosis of bacterial vaginosis.

Although our study was based only on the Papanicolaou test, the frequency of *Candida* (22.5%), *Gardnerella* (15.9%) and *Trichomonas* (3.4%) in the past decade was similar to that reported by Riviera et al.,^13^ who encountered frequencies of 26%, 16.5% and 1.7% for these agents, respectively. These authors studied 405 women, also studying them clinically in relation to the pH of the vaginal secretion and production of amines and, under the microscope, via fresh examination and Gram staining to evaluate the presence of fungi, *Trichomonas* and "clue cells".

In relation to infections being most frequent in patients aged under 20 years, there is a difficulty in making comparisons with data in the literature, as authors have generally not presented details of their analyses according to age group. Nevertheless, in our material, the frequency of *Candida* (30% in 1998) and *Gardnerella* (17.7% in 1998) in patients aged under 20 years was greater than in the material of Carvalho et al.,^16^ who found *Candida* in 19.5% and *Gardnerella* in 14% of the 128 adolescents analyzed. On the other hand, *Trichomonas* was more frequent in the adolescents of the study by Carvalho et al (9.4%)^16^ than in our material (3.2% in 1998).

This study shows the historical evolution of the cytopathology service at Faculdade de Medicina do Triângulo Mineiro, giving evidence of the diagnostic difficulties in the beginning and the changes in frequency of the different infections that possibly are related to living and hygiene habits of the population and the introduction of different medications over these four decades.

## CONCLUSIONS

Over the four decades studied, there has been a decrease in the frequency of cervical-vaginal infection by *Trichomonas vaginalis* and an increase in the frequency of *Candida sp*, especially over the last decade. In a general manner, all the infections were most frequent in patients aged under 20 years and least frequent in patients aged 50 years and over.
